# Supplementary material for: Development of a high throughput system to screen compounds that revert the activated hepatic stellate cells to a quiescent-like state
Source: Sci Rep. 2024 Apr 12;14:8536. doi: 10.1038/s41598-024-58989-6 (PMC11014936; doi:10.1038/s41598-024-58989-6)
Supplement: Supplementary file 1 — Supplementary Information. [file 41598_2024_58989_MOESM1_ESM.pdf]

Supplemental Figures and Legends

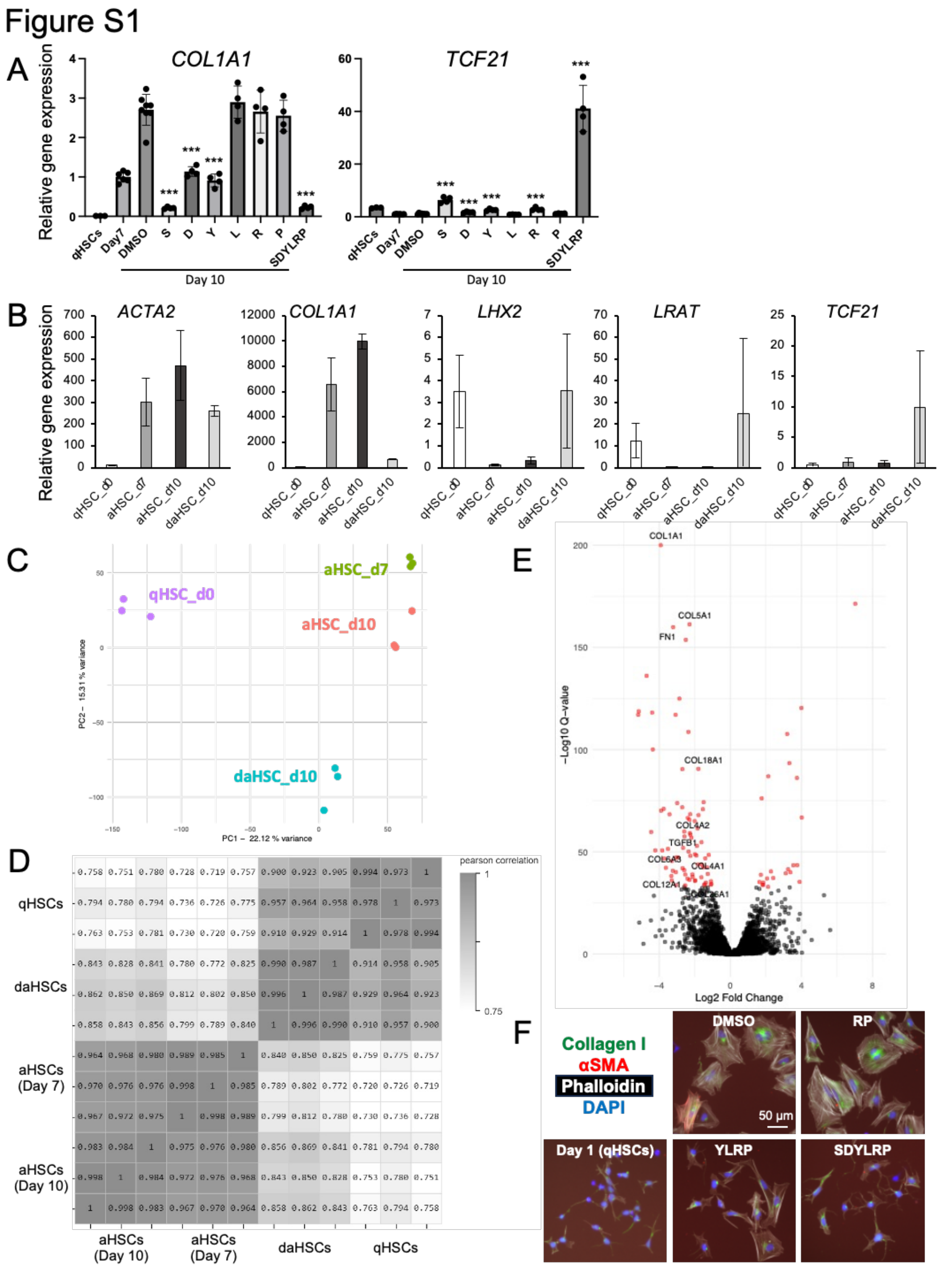

Figure S1. Deactivation of activated HSCs in vitro, related to Figure 1.

(A) Expression levels of *COL1A1* and *TCF21*. The results are shown as the mean ± SD (each

experiment contains three technical replicates). Y: Y27632, L: Lanifibranor, R: Retinoic acid, P: Palmitic acid, S: SB431542, D: Dorsomorphin. The expression level of 7-day cultured aHSCs without DMSO was set to 1. ***P<0.001. (B) Relative gene expression of HSC markers in qHSCs (day 0), aHSCs (day 7 and day 10) and daHSCs. The results are shown as the mean ± SD of independent experiments. (C) Principal Component Analysis (PCA) plot illustrating the variance in gene expression among qHSCs (day 0), aHSCs (day 7), aHSCs (day 10), and daHSCs (day 10), based on TPM values of all expressed genes. (D) Pearson correlation coefficient of gene expression profiles between qHSCs, aHSCs (day 7), aHSCs (day 10) and daHSCs (day 10). (E) Volcano Plot of differential expression analysis between aHSCs (day 10) and daHSCs (day 10). The top 30 significant genes, highlighted in red, are selected based on their Q-value. Labels identify genes associated with fibrosis. Notably, the -Log10 Q-value of COL1A1 is clipped to 200 due to its extremely low value, enhancing visibility within the plot. (F) Fluorescence images (Collagen I: green, αSMA: red and phalloidin: white) of qHSCs (day 0) and aHSCs treated with DMSO, RP, LRP, YLRP, SDLRP or SDYLRP. Y: Y27632, L: Lanifibranor, R: Retinoic acid, P: Palmitic acid, S: SB431542, D: Dorsomorphin. Nuclei were stained with DAPI. Scale bar, 50 μm.

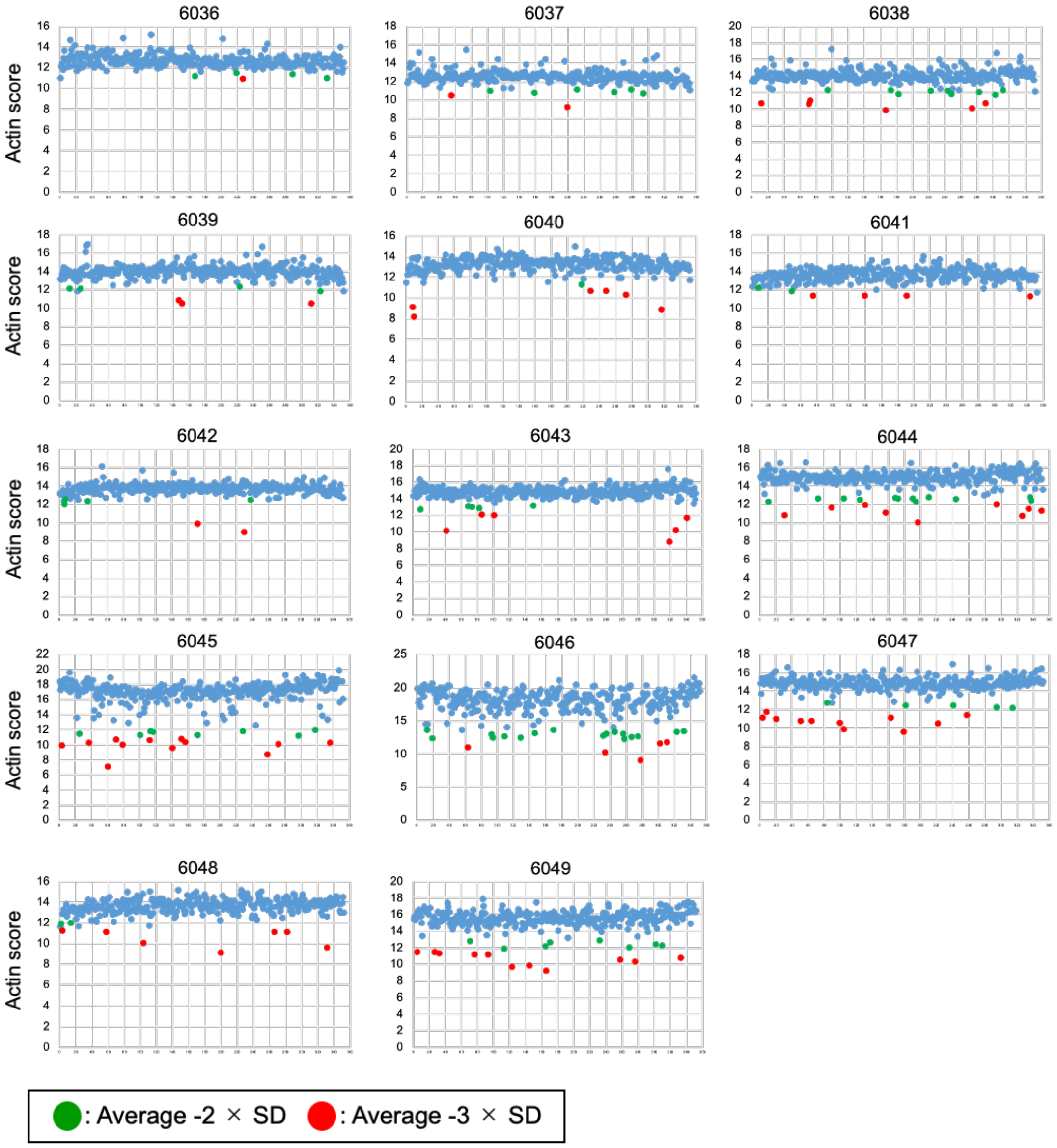

Figure S2. Screening of deactivation chemicals from the validated chemical library, related to Figure 3.

Dot plots of Actin score per well of 384-well plates (plate number 6036 to 6049) incubated with compounds of the chemical library. Red dots indicate compounds with -3 SD of the mean and green dots indicate compounds with -2 SD of the mean.

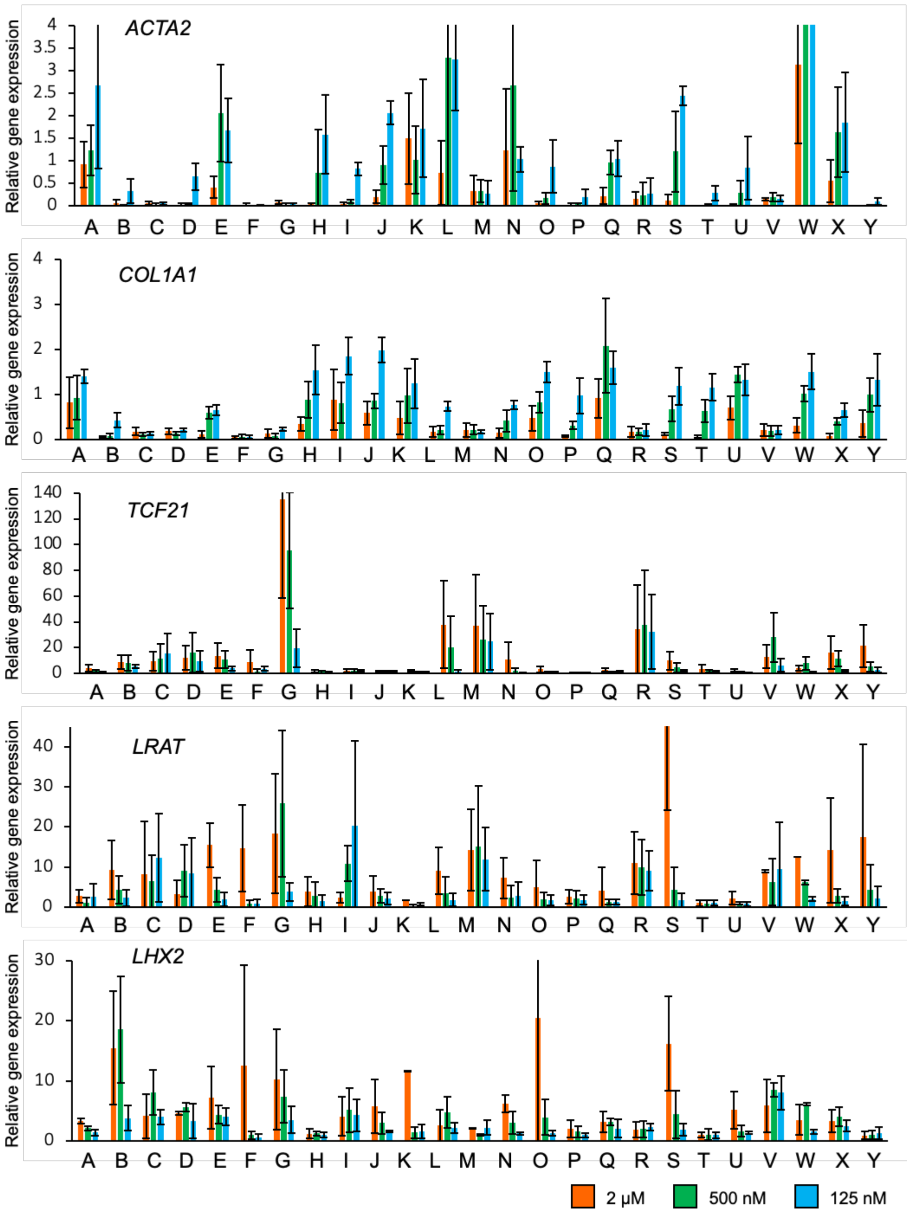

Figure S3. Relative gene Expression levels of HSC markers, related to Figure 3.

Relative expression levels of *ACTA2*, *COL1A1*, *TCF21*, *LRAT* and *LHX2* in hiPSC-derived aHSCs incubated with compound A to Y at the concentration of 125 nM, 500 nM and 2 μM. The values of samples treated with DMSO were set to 1. The results are shown as the mean ± SD of independent experiments (each experiment contains three technical replicates).

Supplemental Tables

Table S1. List of quantitative PCR primers

ACTA2	CAGCCAAGCACTGTCAGG
	CCAGAGCCATTGTCACACAC
COL1A1	AAGAGGAAGGCCAAGTCGAG
	CACACGTCTCGGTCATGGTA
GAPDH	AAGGTGAAGGTCGGAGTCAA
	AATGAAGGGGTCATTGATGG
LHX2	ATGCTGTTCCACAGTCTGTCG
	GCATGGTCGTCTCGGTGTC
LRAT	TACTGCAGATATGGCACCCC
	CCAAGACTGCTGAAGCAAGA
MDK	CAAGTTTGAGAACTGGGGTGCGTG
	AGTCCTTTCCCTTCCCTTTCTTGG
PTN	CAGCGTCGAAAATTTGCAGCTGC
	TCCACTGCCATTCTCCACAGTCAG
TCF21	CACTTGAGGCAGATCCTGGCTA
	CGGTCACCACTTCTTTCAGGTC
NGFR	CTGCTGCTGTTGCTGCTTCT
	CAGGCTTTGCAGCACTCAC
